# Efficacy and safety of autologous or allogeneic mesenchymal stromal cells from adult adipose tissue expanded and combined with tricalcium phosphate biomaterial for the surgical treatment of atrophic nonunion of long bones: a phase II clinical trial

**DOI:** 10.1186/s12967-024-05280-x

**Published:** 2024-05-24

**Authors:** Lluís Orozco Delclós, Robert Soler Rich, Rafael Arriaza Loureda, Alonso Moreno García, Enrique Gómez Barrena

**Affiliations:** 1 Institut de Teràpia Regenerativa Tissular, Centro Médico Teknon, Barcelona, Spain; 2 Instituto Médico Arriaza, Grupo INCIDE Universidad da Coruña, A Coruña, Spain; 3 Department of Orthopaedic Surgery and Traumatology, Hospital Universitario La Paz-IdiPaz, Madrid, Spain; 4 School of Medicine, Universidad Autónoma de Madrid, Madrid, Spain

**Keywords:** Bone graft, Atrophic nonunion, Bone marrow mesenchymal stem cells, Adipose tissue mesenchymal stromal cells, Long bone, Fracture

## Abstract

**Background:**

Autologous bone grafting is the standard treatment for the surgical management of atrophic nonunion of long bones. Other solutions, such as bone marrow mesenchymal stem cells (BM-MSC) combined with phospho-calcium material, have also been used. Here we evaluate the safety and early efficacy of a novel procedure using autologous or allogenic adipose tissue mesenchymal stromal cells (AT-MSC) seeded in a patented tricalcium phosphate-based biomaterial for the treatment of bone regeneration in cases of atrophic nonunion.

**Methods:**

This was a prospective, multicentric, open-label, phase 2 clinical trial of patients with atrophic nonunion of long bones. Biografts of autologous or allogenic AT-MSC combined with a phosphate substrate were manufactured prior to the surgical procedures. The primary efficacy was measured 6 months after surgery, but patients were followed for 12 months after surgery and a further year out of the scope of the study. All adverse events were recorded. This cohort was compared with a historical cohort of 14 cases treated by the same research team with autologous BM-MSC.

**Results:**

A total of 12 patients with atrophic nonunion of long bones were included. The mean (SD) age was 41.2 (12.1) years and 66.7% were men. Bone healing was achieved in 10 of the 12 cases (83%) treated with the AT-MSC biografts, a percentage of healing similar (11 of the 14 cases, 79%) to that achieved in patients treated with autologous BM-MSC. Overall, two adverse events, in the same patient, were considered related to the procedure.

**Conclusions:**

The results of this study suggest that AT-MSC biografts are safe for the treatment of bone regeneration in cases of atrophic nonunion and reach high healing rates.

**Trial registration:**

Study registered with EUDRA-CT (2013-000930-37) and ClinicalTrials.gov (NCT02483364).

**Supplementary Information:**

The online version contains supplementary material available at 10.1186/s12967-024-05280-x.

## Background

About 2.2 million bone grafting procedures are performed annually worldwide [1]. Patients with atrophic nonunion (fractures which do not heal within 8 months, characterized by the absence of “bone callus” and the interposition of fibrous tissue between both segments) often require autologous bone grafting, as this treatment provides an osteoconductive structure, progenitor cells, morphogenic proteins, and growth factors that induce angiogenesis and osteogenesis [2]. These elements, together with mechanical stability, are essential for the healing of fractures [3]. During the past decade various surgical approaches have been tested to treat atrophic nonunions, although there is no consensus on which one is the best technique for any specific situation [4–9]. Autologous cancellous bone grafting is still considered the ‘gold standard’ [10–12], but the process of obtaining the autologous graft increases the surgery time and has an associated morbidity, such as persistent pain in the donor area [13, 14]. The iliac bone is usually the autologous bone graft donor site, but it can only provide a limited volume of tissue, especially if it has been previously used [15]. To solve the inconveniences of the surgical procedure and the low content of progenitor cells of autologous bone grafting, other alternatives such as allogeneic, cryopreserved, or lyophilized bone have been used. These options, although acellular, have osteoconducting properties and carry osteoinductive molecules.

Some clinical trials have focused on the application of mesenchymal stromal cells (MSCs) or MSC-based bone tissue on bone regeneration [16, 17]. Systems based on processing and enrichment of autologous or allogenic progenitor cells could potentially achieve bone regeneration at the nonunion site in a shorter time, avoiding the risks and disadvantages of graft-obtaining surgery. Recently, a new therapeutic alternative was developed consisting of an osteoconductive structure colonized by high doses of cultured adipose tissue-derived mesenchymal stromal cells (AT-MSC) pre-differentiated to the osteoblastic line. Adipose tissue is considered an easy-to-access tissue and can be obtained with a low incidence of comorbidity. AT-MSC have a multi-lineage potential that is very similar to BM-MSC [18, 19], have been found to have similar expression of phenotypic markers [20]. However, in some patients it is not possible to obtain sufficient adipose tissue to have an adequate amount of AT-MSC with therapeutic potential (in the differentiation phase and releasing secretome). The alternative to an autologous graft procedure is the use of cryopreserved banks of allogeneic AT-MSC from adipose tissue of healthy donors. This alternative system based on biomaterial designed for bone regeneration purposes, totally recovered with allogenic AT-MSC, would provide a tissue-engineering biograft product with which to accelerate the formation of new and stable bone at the site of the nonunion. The use of this system would avoid the disadvantages of surgery for obtaining the graft. Here we present the results of a multicenter phase II trial to evaluate this approach and the feasibility and safety of a novel biograft product based on these principles in humans.

## Materials and methods

This was a prospective, multicentric, open-label, phase 2 clinical trial for the surgical treatment of atrophic nonunion of long bones using adult autologous or allogeneic AT-MSC associated with tricalcium phosphate biomaterial. See Supplementary Materials for brief additional information on the pre-clinical studies on which this study was based. The study was conducted from 2 June 2015 to 20 October 2020 at the Institut de Teràpia Regenerativa Tissular (Centro Médico Teknon, Barcelona, Spain) and the Hospital Universitario La Paz (Madrid, Spain). The trial followed the criteria of the Osteosynthesis Association for the Study of Internal Fixation (AO-ASIF) and was authorized by the Ethics Committee of the Quirón Salud Hospital Group (protocol STEMQUIRI/12ES01, on 24 May 2018) and the Spanish Agency for Medicines and Health Products (AEMPS). Informed consent was obtained from all patients. The trial was conducted in accordance with the US Health Insurance Portability and Accountability Act (HIPAA) and the Declaration of Helsinki. It was registered with EudraCT number as 2013-000930-37 and with ClinicalTrials.gov as NCT02483364.

### Patients

Subjects were recruited between 18 and 65 years of age of both sexes, diagnosed with radiographically confirmed atrophic nonunion of a long bone. Patients were excluded if presenting any infection, other lesions interfering with weight bearing, open pseudoarthrosis at the time of inclusion, congenital bone diseases (hypophosphatemia), metabolic bone disease associated with primary or secondary hypoparathyroidism, or other conditions or circumstances according to medical criteria.

### Study procedures

See Supplementary Methods for full details of scheduled visits applicable to the 6 patients treated with the autologous (Table S1) and the 6 patients treated with allogeneic (Table S2) biografts. For patients treated with the autologous biograft product an outpatient surgery visit was arranged for the aspiration of adipose tissue plus biograft application surgery 36 days later (time required for isolation, expansion and seeding the cells in the biomaterials, final product preparation and referral to the hospital). Patients who were treated with the allogeneic biograft product did not have the outpatient surgery visit. There was a period of 15–18 days from the screening visit to the visit for the surgical implantation (time required to process the product and have the appropriate dose of biograft). Once they have had the surgery, the patients were followed up for safety and efficacy (by way of X-rays) as established in the visit schedule (Tables S1 and S2).

### Autologous AT-MSC processing

The AT-MSC were obtained from 100 mL of lipoaspirate from the abdominal wall obtained under local anesthesia and sedation. The cells were processed under controlled temperature conditions (2–6ºC) at the Histocell laboratories (Derio, Spain) under good manufacturing practice (GMP) standards for clinical application. The AT-MSC were isolated through an enzymatic digestion process and their number amplified using the usual media in cell culture processes. When the culture in passage 3 reaches semiconfluency, a specific osteogenic pre-differentiating medium (STEMPRO® Osteogenic Differentiation Kit; Gibco Life Technologies) was added to the cells and the culture maintained for 8 days.

### Allogeneic AT-MSC processing

All the implanted allogeneic cells derived from a Master Cell Bank (MCB) of MSC obtained from the same donor (See Supplementary Methods for details on donor selection, and Table S3). The manufacturing process included the generation of a cryopreserved working cell bank (WCB) at passage 3. Once the patient was recruited and surgery scheduled, enough cells for product manufacturing were thawed, and once reached semiconfluency, cells were pre-differentiated for 8 days using specific GMP culture media (STEMPRO® Osteogenic Differentiation Kit, Gibco Life Technologies) (See Supplementary Methods for determination of differentiation time).

### Final product preparation and quality control

The pre-differentiated cells were seeded on cylindrical matrices (CMT) 3 to 5 mm in size and kept for 4 days to ensure adherence. The matrices were composed of β-tricalcium phosphate (F 1088 standard specification for β-tricalcium phosphate for surgical implantation, Sigma-Aldrich), monobasic calcium phosphate (Budenheim Aldrich), calcium carbonate (Ph. Eur. Specifications, Sigma-Aldrich) and sodium pyrophosphate authorized as a food additive by the FDA (BK Giulini GmbH Aldrich). The porosity of the biomaterial was adequate for the colonization of each CMT with 4 ± 0.8 × 10^5^ AT-MSC. Pre-differentiated AT-MSCs maintained the phenotypic and immunophenotypic characteristics required by the criteria defined by the International Society for Cellular Therapy for MSC: (1) in vitro adherent fibroblastoid morphology; (2) Expression of the CD105, CD73 and CD90 antigens with the absence of hematopoietic markers such as CD45, CD34, CD14 or CD11b, CD79 or CD19 and HLA-DR; and (3) Ability to differentiate in vitro into osteoblasts, adipoblasts, and chondroblasts (See Supplementary Methods for details on characterization) [21]. Subsequent steps included determining the immunophenotype and the viability of the cells in their final container (See Supplementary Materials Table S4 for immunophenotypic characterization and support adhesion of batches used), carrying out genetic stability tests, calculating the total number of generations and ensuring the microbiological quality by means of sterility, mycoplasma, bacterial endotoxin and GRAM control tests. The cells were supplied to the surgical team ready for application in syringes containing 10 CMT in a gelled medium (Ringer’s lactate, 82.8%; Gelita-Spon® gelatine, 5.7%; 33 g/L glucosaline solution, 2.9%; 1.4% sodium bicarbonate, 8.6%) which ensures the viability of the cells that colonize the interior and exterior of the biomaterial for which they show great affinity. Each syringe with 10 CMT provided a dose of 4 ± 0.8 × 10^6^ AT-MSC (See Fig. S1). The total dose to be used was determined by the volume of the lesion (Table S5). A representative example of the cellular response in the matrix over time is shown in Fig. S2. This product is patented (PCT/ES2009/000358) by Histocell SL (Spain).

### Endpoints and assessments

The primary objective of this study was to assess safety of the intervention and, additionally, the efficacy by radiographic criteria within 6 months after surgery. The assessment included evaluation of the clinical evolution of the lesion, distal trophism, vascular status, healing, joint balance, comparative measurement of muscle circumference in both legs or arms, deviations, and rotations. The evolution of the lesion was determined by radiographs in frontal and profile projections and computed tomography scans were conducted only when necessary to avoid unnecessary exposure given the accumulation of previous radiographic examinations of the patients in the trial. The radiographs were consistently assessed for efficacy by the surgeon following the accepted radiographic criteria. The efficacy of the intervention using radiographic criteria was determined by evidence of “callus” formation in 3 of 4 cortices (humerus, ulna, radius or femur) or 2 of 3 cortices (tibia) within 6 months of surgery. The variable used was the time in weeks from surgery to “callus” formation determined using radiographic criteria for fracture healing. Any adverse events (AEs) occurring during the conducting of the trial, either observed by the investigator or reported by subjects themselves, were recorded.

The secondary objective was the comparison of the incidence of AEs, complications, and healing times, between the 12 patients in this study and the data from a historic cohort of 14 consecutive cases of refractory long bone nonunion who have been treated since 2009 with a fixed cell dose of 40 × 10^6^ autologous bone marrow MSC (BM-MSC), expanded at the Institute of Molecular Biology and Genetics (IBGM) of Valladolid (Spain), corresponding to authorized compassionate use treatments, and subject to AEMPS control after the clinical trial on nonunion treated with stem progenitor cells (registered with EUDRA-CT number 2005-001755-38). The BM-MSC were included in 3–5 mm chips of lyophilized allogeneic cancellous bone in a quantity adjusted to the specific treatment and the whole included in an autologous fibrin clot. To comply with the characteristics required by the International Society for Cell & Gene Therapy [21], they were delivered to the surgical team in a suspension of lactated Ringer, 0.2% human albumin and 5 mM glucose. The components were mixed in the operating room just prior to implantation.

## Results

A total of 12 patients with atrophic nonunion were included in the trial **(**Table 1**)**. Of the 12 patients, 8 (66.7%) were men. Mean (± SD) age was 41.2 ± 12.11 years with a range of 19 to 58 years. On average the patients had had 1.9 surgeries prior to the inclusion to the study. The most common site of the lesion for the randomized patients overall was the tibia (41.7%); the rest of locations were femur and humerus with a 16.7% each, and ulna, clavicle and first metatarsal with an 8.3% each. Autologous AT-MSC were applied in the first 6 cases treated, which involved a first stage consisting of cell obtention through liposuction. Allogeneic AT-MSC were used in the other 6 cases. The maximum applied cell dose was 56 × 10^6^ AT-MSC in a femur and the lowest 6 × 10^6^ AT-MSC in a metatarsal.

**Table 1:** Patients (*N* = 12) and status at 6 months

Patient	Sex	Age	Bone treated	Prior surgery	Cell dose	Status at 6 months		
						Callus formation	Number of bridges	Fracture status
Autologous AT-MSC								
1-01-01	M	39	Femur	1	56 × 10^6^	Yes	4	Healed
1-02-02	M	47	Tibia	1	24 × 10^6^	Yes	4	Healed
1-04-03	M	19	Femur	3	33 × 10^6^	Yes	4	Healed
1-05-04	F	58	First metatarsal	2	6.4 × 10^6^	Yes	2	Healed
1-06-05	M	45	Tibia	3	12 × 10^6^	Yes	-^1^	Healed
2-01-01	M	49	Tibia	2	15 × 10^6^	Yes	2	Healed
Allogeneic AT-MSC								
1-07-06	F	35	Clavicle	1	12 × 10^6^	Yes	2	Healed
1-08-07	M	38	Tibia	3	40 × 10^6^	Yes	2	Healed
1-11-08	M	22	Ulna	1	16 × 10^6^	Yes	3	Healed
1-12-09	F	52	Humerus	4	32 × 10^6^	No	-	Not healed
1-14-10	F	54	Tibia	2	39 × 10^6^	Yes	3	Healed
2-02-02	M	36	Humerus	1	12 × 10^6^	Yes	1	Not healed

^1^ Due to the characteristics of the lesion it was not possible to verify the number of bridges

Abbreviations: AT-MSC, adipose tissue mesenchymal stromal cells; F, female; M, male

Safety was evaluated throughout the 12 months of study duration, with a total of 36 AEs reported (Table 2). Only 2 of them were considered related to the procedure (2 cases of device expulsion in the same patient). One patient withdrew from the study after 9 months due a non-related serious AE (device loosening). No other serious AE was observed in any study patient. In the cases involving liposuction, the patients tolerated the procedure well in terms of pain and the only remarkable AE was a diffuse ecchymosis along the abdominal wall that persisted for a few weeks. In the case of allogenic biografts, the designed procedure was shown to be viable and no relevant AEs attributable to the biografts were observed.

**Table 2:** Adverse events observed in this study

	Total (*N* = 12)		Autologous AT-MSC (*N* = 6)		Allogenic AT-MSC (*N* = 6)	
	Number of patients, *n* (%)	Number of events	Number of patients, *n* (%)	Number of events	Number of patients, *n* (%)	Number of events
**AEs**	11 (91.7)	36	6 (100)	19	5 (83.3)	17
Infections and infestations	4 (33.3)^a^	5	2 (33.3)	3	2 (33.3)	2
Psychiatric disorders	2 (16.7)^b^	3	1 (16.7)	2	1 (16.7)	1
Nervous system disorders	1 (8.3)	2	1 (16.7)	2	0	0
Vascular disorders	1 (8.3)	1	0	0	1 (16.7)	1
Respiratory, thoracic and mediastinal disorders	1 (8.3)	1	0	0	1 (16.7)	1
Gastrointestinal disorders	1 (8.3)	1	1 (16.7)	1	0	0
Skin and subcutaneous tissue disorders	2 (16.7)	2	2 (33.3)	2	0	0
Musculoskeletal and connective tissue disorders	3 (25.0)	4	2 (33.3)	3	1 (16.7)	1
General disorders and administration site conditions	6 (50.0)	9	3 (50.0)	5	3 (50.0)	4
Injury, poisoning and procedural complications	3 (25.0)	3	0	0	3 (50.0)	3
Surgical and medical procedures	1 (8.3)	1	0	0	1 (16.7)	1
Product issues	3 (25.0)	4	1 (16.7)	1	2 (33.3)	3
**Related AEs**	1 (8.3)	2	0	0	1 (16.7)	2
Product issues	1 (8.3)	2	0	0	1 (16.7)	2
**Serious AEs**	1 (8.3)	1	0	0	1 (16.7)	1
Product issues	1 (8.3)	1	0	0	1 (16.7)	1
**Serious related AEs**	0	0	0	0	0	0

No AEs were reported for which the outcome was ‘Death’ or ‘Resolved with sequelae’

Percentages were calculated with respect to total patients

Abbreviations: AE, adverse event; AT-MSC, adipose tissue mesenchymal stromal cells

^a^ Includes 5 events related to 4 patients (abscess, bronchitis, cystitis, *Staphylococcus* test and a viral infection). None of the events were related to the study medication

^b^ Includes 3 events in 2 patients (2 episodes of anxiety treated with fluoxetine and alprazolam, and 1 of insomnia, treated with melatonin). None of the events were related to the study medication

**Table 3:** Historical cohort (*N* = 14). Clinical characteristics and results of the treatment with BM-MSC. The cell dose was fixed and always 40 × 10^6^ cells

Patient	Sex	Age	Bone treated	Prior surgery	Cell dose	Fracture status	Related AEs
14	F	20	Tibia	1	40 × 10^6^	Healed	No
15	M	22	Tibia	1	40 × 10^6^	Healed	No
16	M	36	Femur	1	40 × 10^6^	Healed	No
17	M	50	Clavicle	4	40 × 10^6^	Not healed	No
18	F	63	Femur	5	40 × 10^6^	Healed	No
19	M	54	Tibia	1	40 × 10^6^	Not healed	No
20	M	30	Humerus	7	40 × 10^6^	Healed	No
21	F	59	Femur	2	40 × 10^6^	Healed	No
22	M	34	Femur	3	40 × 10^6^	Healed	No
23	F	43	Tibia	2	40 × 10^6^	Healed	No
24	M	35	Tíbia	3	40 × 10^6^	Healed	No
25	M	59	Tibia	2	40 × 10^6^	Healed	No
26	M	42	Femur	2	40 × 10^6^	Not healed	No
27	M	64	Femur	2	40 × 10^6^	Healed	No

Abbreviations: AEs, adverse events; BM-MSC, bone marrow mesenchymal stromal cells; F, female; M, male

**Fig. 1:**
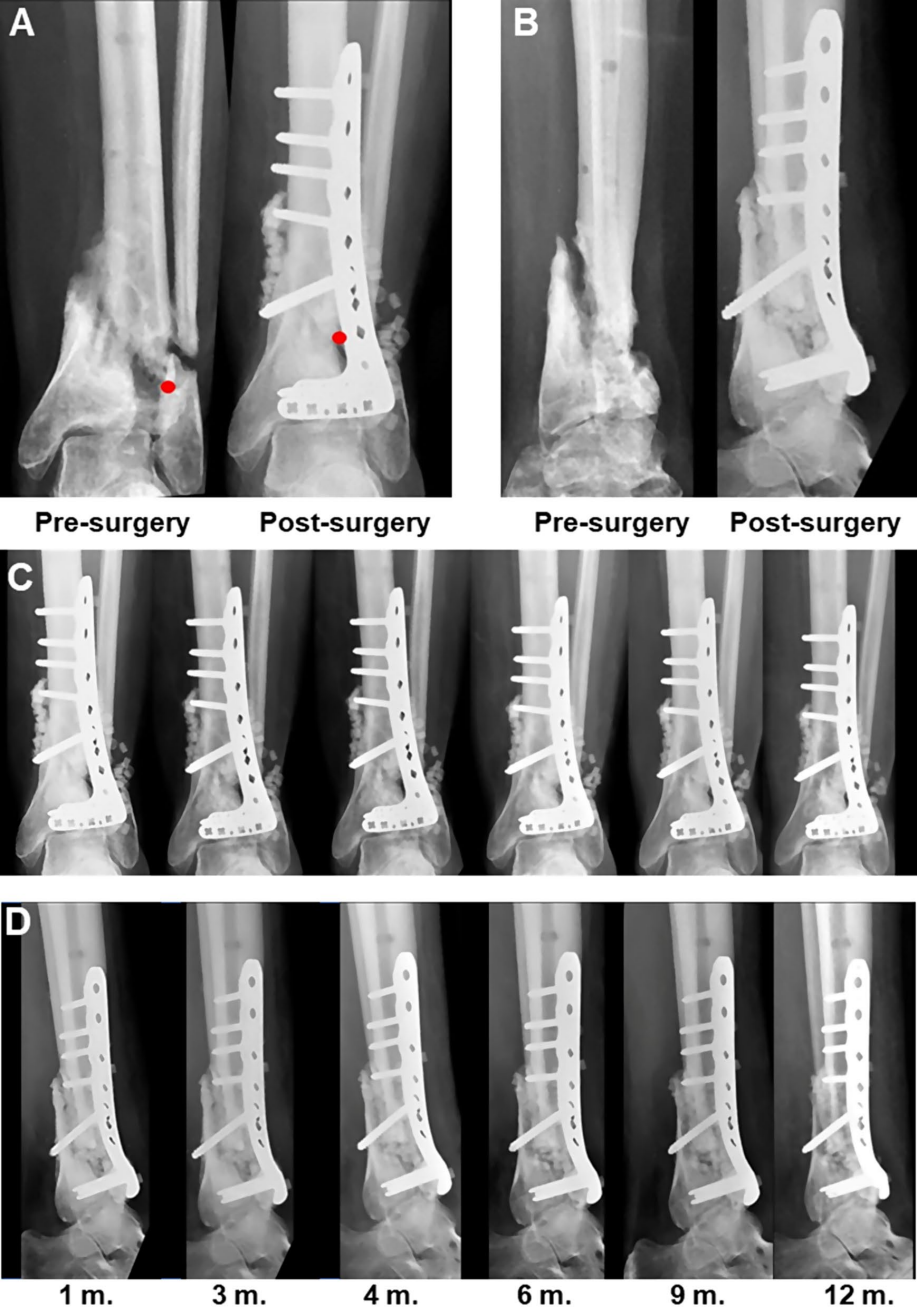
Example of use of allogeneic AT-MSC in a tibia nonunion (patient 1-14-10). X-ray anteroposterior (**A** and **C**) and lateral projections (**B** and **D**). **A** and **B** show fracture before and after surgery; **C** and **D** show the follow up until month 12. The 54-year-old female patient initially presented an open tibial pilon fracture treated with external fixation and minimal osteosynthesis with Kirschner wires. Sepsis was confirmed three weeks later, which was treated with debridement, circumferential fixation, and antibiotics. After three months the external fixation was withdrawn and replaced by a cast, and subsequently by a removable orthosis without load. The fracture evolved into a nonunion. Thirteen months after the initial fracture, and after ruling out a septic process, curettage of the wound was conducted. Once an acceptable anatomical realignment had been achieved, a large space lost bone was observed inside the metaphysis; the contact area between segments was very limited and only on the internal slope of the tibia. Stability was achieved with a lag screw and an anterolateral distal tibial plate overlying it. An allogenic biograft consisting of 98 monetites was applied, with a total dose of 39 × 10^6^ AT-MSC. The patient presented a good postoperative course, without inflammatory signs, and was discharged from the hospital after 4 days without immobilization. Healing was verified at 6 months, with gait being painless and presenting only limited joint balance in dorsiflexion (5º)

The main efficacy variable was bone healing, assessed by radiographic criteria within 6 months after surgery, and was achieved in 10 of the 12 treated cases (83%). A representative example of a case treated with allogenic AT-MSC is shown in Fig. 1.

The historical cohort of 14 consecutive cases was treated by the same research group with a fixed cell dose of 40 × 10^6^ MSC from autologous bone marrow mesenchymal stromal cells (BM-MSC) **(**Table 3**)**. In this cohort, bone healing was observed in 11 of the 14 treated cases (79%).

The study included an additional 24-month safety visit requested by the regulatory agency. During this period, one non-related AE (removal of two long screws of the osteosynthesis) and one non-related serious AE (tibial fracture) were observed. This last case was considered radiographically healed at the 6-month visit, but it could have been a misinterpretation of the diagnosis of healing or a refracture due to the fragility of the achieved bone callus. These 2 AEs do not change the global safety conclusions of the study.

## Discussion

The results from this phase 2 study suggest that the treatment of atrophic nonunion of long bones by means of cultured MSC derived from autologous or allogeneic fat cells is safe and offers an efficacy rate comparable to that achieved with surgical techniques involving other techniques, such as the use of cultured BM-MSC. With this alternative system based on the amplification in culture of AT-MSC and its inclusion in biomaterial the drawbacks of surgery to obtain bone autograft were avoided.

In this study we used autologous and allogenic AT-MSC biografts. The manufacturing process of the allogeneic bone obtained from a healthy donor was identical to the autologous one. The difference lies in the use of a MCB and WCB from the same donor that includes cryopreservation and thawing steps before final product preparation, which was not carried out for the autologous product. The obvious advantage of using allogeneic MSC is that it spared for the patient the surgical procedure associated with the adipose tissue sample obtention, performed under anesthesia, and the 36-day treatment delay, which is the time invested in the production of the biograft. As the autologous AT-MSC biografts require considerable time before they are available for use, they are not suitable for acute diseases or those requiring rapid treatment [22]. The alternative use of allogeneic AT-MSC, obtained from healthy donor adipose tissue and cryopreserved, overcomes these problems. In either case, the autologous or the allogeneic AT-MSC biografts contain a high dose of progenitor cells. Also, in the case of allogenic biografts, the possibility of an immune reaction is reduced, as it has been widely demonstrated that MSC evade antigenic recognition and also inhibit immune responses, being tolerated even if the cells are intravenously administered at a large dose. Due to their immunomodulatory potential, AT-MSC have been applied in graft versus host disease or autoimmune diseases, including against the cytokine storm linked to SARS-CoV-2 infection [23].

The procedure to obtain autologous adipose tissue can be more complex in elderly patients or patients with a low body fat index, or in patients with diabetes, rheumatoid arthritis, or systemic lupus erythematosus.

The autologous or allogenic AT-MSC were included in a biomaterial whose composition and form was designed to exert a retentive effect on the cells and facilitate their proliferation. The biomaterial was suspended in a gelatinous solution with compacting effect and supplied to the surgeons in a ready-to-use syringe. Therefore, it did not require any additional manipulation during surgery, except for appropriate distribution in the nonunion site treated, avoiding excessive compaction that would compromise cell viability and the conductivity and revascularization of the biograft.

The structure and composition of the biomaterial are key factors to achieve bone regeneration. Unlike TCP granules already on the market, monetite (TCP derivative) interacts better with host bone cells and their physical structure includes homogeneously distributed microporosity and transversal macro channels that go across entire cylinders and that are totally colonized by MSC. This strategy provides a better interaction in the implanted area, creating a microenvironment that facilitates new tissue revascularization [24, 25].

The comparison between the cases treated with AT-MSC and the historical series treated by the same surgical team with a fixed dose of 40 × 10^6^ autologous BM-MSC revealed a similar rate of efficacy and safety, even though a significantly lower cell dose was implanted in 9 of the 12 cases. Positive results were also obtained in the international, non-comparative multicenter clinical trial that included 28 patients in which much higher cell doses (100 to 200 × 10^6^) of autologous BM-MSC were used in 5–10 cc of phospho-calcium material [26]. The variability of the cases, protocols and surgical procedures preclude any precise comparison between the present work and other pilot studies, but favorable results may be obtained by different techniques. Hernigou reported good results by simply inoculating a bone marrow concentrate at the focus of the atrophic nonunion of the tibia without further surgery such as osteosynthesis or even the replacement of osteosynthesis material, which are necessary actions in most nonunion cases [27].

One of the limitations of this study is the small sample size and the heterogeneity in the distribution of cases, but this is a common problem in early studies that treat a condition such as atrophic nonunion that involves a surgical intervention.

## Conclusions

The described procedure using both autologous and allogeneic AT-MSC, pre-differentiated to the osteoblastic line and incorporated into special designed calcium phosphate-based biomaterials, suggests that it is safe for use in humans for the treatment of atrophic nonunion. A major advantage of this technology is to avoid a bone graft, which is the standard therapy for long bone nonunion. We believe that the results from this clinical trial also confirm the feasibility of manufacturing and delivering AT-MSC-based biografts. The analysis of the adverse effects reported suggest that their use is safe in cases requiring bone grafting. Taken together, the results suggest that further studies are needed to explore of the efficacy of the procedures described.

### Electronic supplementary material

Below is the link to the electronic supplementary material.

Supplementary Material 1

## Data Availability

The datasets supporting the findings of this study are available from the corresponding author upon reasonable request.
