# Supplementary material for: Efficacy and safety of autologous or allogeneic mesenchymal stromal cells from adult adipose tissue expanded and combined with tricalcium phosphate biomaterial for the surgical treatment of atrophic nonunion of long bones: a phase II clinical trial
Source: J Transl Med. 2024 May 24;22:493. doi: 10.1186/s12967-024-05280-x (PMC11127443; doi:10.1186/s12967-024-05280-x)

**- SUPPLEMENTARY MATERIALS -**

**Efficacy and safety of Autologous or Allogeneic Mesenchymal Stromal Cells from Adult Adipose Tissue Expanded and Combined with Tricalcium Phosphate Biomaterial for the Surgical Treatment of Atrophic Nonunion of Long Bones: A Phase II Clinical Trial**

Lluís Orozco Delclós^1^, Robert Soler Rich^1^, Rafael Arriaza Loureda^2^, Alonso Moreno García^3^, Enrique Gómez Barrena^3,4^

^1^ Institut de Teràpia Regenerativa Tissular, Centro Médico Teknon, Barcelona, Spain
^2^ Instituto Médico Arriaza, Grupo INCIDE Universidad da Coruña, A Coruña, Spain
^3^ Department of Orthopaedic Surgery and Traumatology, Hospital Universitario La Paz-IdiPaz, Madrid, Spain

^4^ School of Medicine, Universidad Autónoma de Madrid, Madrid, Spain

Correspondence: Lluís Orozco Delclós, lluis.orozco@itrt.es

**Supplementary Methods**

**Preclinical *in vitro* and *in vivo* studies**

This approach described in this study was tested in several preclinical *in vitro* and *in vivo* studies (not published). Safety results on ploidy, chromosomal stability and substrate fixation studies showed good adequacy of the technique, which was also confirmed through *in vivo* ectopic studies. Efficacy was also evaluated in CD1 nude mice, showing higher bone regeneration activity compared to the biomaterial alone and the biomaterial with non-pre-differentiated cells. Additionally, a bone healing study within a critical sized bone defect in ovine iliac crest performed in AO Foundation, Davos (Switzerland), demonstrated increased bone formation when associating pre-differentiated ASC to the biomaterial vs. the biomaterial alone. One further study assessing the biodistribution, tumorigenic potential and efficacy was conducted in immunosuppressed rats. The study concluded that the product is effective in locomotor functional recovery and bone regeneration, and no concerns or alterations were observed regarding all the evaluated safety aspects.

**Selection of the donor of allogeneic AT-MSC**

The implanted allogeneic cells derived from a Master Cell Bank (MCB) of MSC were obtained from a unique donor. The first step to obtaining the adipose tissue sample was to select the appropriate healthy allogeneic donor. For donor selection, the following inclusion/exclusion criteria are considered (See **Table S3**):

- The candidate will fill in a questionnaire to evaluate the health and medical history of the potential donor. This questionnaire will be administered by the surgeon responsible for the intervention.

- Once the donor is accepted, he/she should be informed about the surgical operation and the aim of the tissue donation before signing the informed consent form.

The donor had serological tests before the surgical operation (7-15 days before the operation and on the day of the operation) to ensure the absence of adventitious agents. For this, a blood sample from the donor was sent to the selected analytical laboratory to confirm the suitability of the donor.

**Determination of differentiation time**

The proposed investigational drug is indicated for bone lesions, so in its development the strategy of in vitro osteogenic predifferentiation of MSCs is adopted for 8 days, so that once the product is implanted in the patient, cell osteogenic differentiation is completed.

Cellular studies have been carried out to analyze that MSCs, after undergoing the described predifferentiation, present initial metabolic characteristics typical of bone cells without losing their pluripotent capacity and, therefore, without ceasing to be normal mesenchymal cells. This osteogenic predisposition of MSCs was analyzed by determining the Alkaline Phosphatase of the cells after 8 days of contact with the specific medium.

The formation of pre-osteoblasts (immature osteoblasts), the initial stage of osteogenic differentiation, is characterized by alkaline phosphatase (ALP) activity. This enzyme participates in the release of inorganic phosphorus from phosphate esters, therefore it intervenes in the mineralization of bone and the maturation of the osteoid matrix. The MSCs differentiated towards bone present high levels of ALP at 4 days and maximum levels at 12 days after osteogenic induction, so ALP activity is used as an indicator of the osteogenic process.

The results have shown that after the selected predifferentiation time, 8 days, an increase in ALP activity was observed in pre-differentiated cells in specific predifferentiation medium with respect to control cells.

**Assays of MSC characterization**

The characterization of the cells was carried out by analyzing the following qualities. The cell batches used for the clinical study complies the assays.

**Adhesion to the support**

The adherence of cells to the support is one of the identification criteria of mesenchymal cells. In this sense, the ability of MSCs to adhere to the plastic support as well as the potential to expand on this support was demonstrated by visualizing the cells in the optical microscope during manufacturing process.

**Characterization by specific cell membrane markers: Immunophenotype**

Characterization of MSCs was carried out by determination of specific surface antigen expression. Immunostaining technique followed by flow cytometry analysis is used to characterize cells. The specific surface antigens analysed and the required specifications as measured by flow cytometry were as follows:

- Positive markers (≥90%) : CD105, CD90, CD44, CD13, CD73

- Negative markers (≥90%): CD19, CD34, CD45, CD14, HLA-DR

***Clonogenic colony forming unit-fibroblast (CFU-F)***

The selected technique to evaluate cell potency in vitro was the quantitative analysis of CFU-F, as one characteristic of the cells is their capacity to generate colonies. Therefore, the efficiency in which this cell type generated colonies in vitro is related with their condition and the maintenance of their essential characteristics. Data obtained from the CFU assay gave quantitative information on the cell product (number of colonies) as well as qualitative information (altered appearance or colony sizes). CFU-F counting was carried out to calculate the percentage efficiency of the studied cells in forming colonies. The specification of the assay is CFU ≥ 20%.

**Differentiation capacity**

MSCs are multipotent cells with the capacity to differentiate into other cell types such as adipocytes, chondrocytes and osteocytes. In this sense, MSCs were characterized by analyzing their differentiation into bone, chondrocytes and adipocytes, determined qualitatively.

**Supplementary Tables**

**Table S1.** Schedule of visits applicable to the patients treated with the autologous biograft product.

|  | **Pre-inclusion** | **Pre-surgery** | **Surgery** | | **Follow-up** | | | | | | |  |
| --- | --- | --- | --- | --- | --- | --- | --- | --- | --- | --- | --- | --- |
|  |  | **V0**  **max. 60 d**  **before V1** | **V1**  **(d 0)** | **V2**  **(+36 d)** | **V3** | **V3’** | **V4** | **V5** | **V6** | **V7** | **V8** |  |
|  |  |  |  | **Surgery** | **1 w** | **1 m** | **3 m** | **4 m** | **6 m** | **9 m** | **12 m** | **24 m*** |
| **Patient information + ICF**  **Request for examinations** | X |  |  |  |  |  |  |  |  |  |  |  |
| **Incl. and excl. criteria** |  | X |  |  |  |  |  |  |  |  |  |  |
| **Medical history** |  | X |  |  |  |  |  |  |  |  |  |  |
| **Physical examination** |  | X |  |  | X | X | X | X | X | X | X | X |
| **Vital signs** |  | X |  |  | X | X | X | X | X | X | X | X |
| **Haematology & clinical biochemistry** |  | X |  |  | X |  |  |  |  |  | X | X |
| **Serology** |  | X |  |  |  |  |  |  |  |  |  |  |
| **Sample for serum bank** |  |  |  | X |  |  |  |  | X |  | X | x |
| **Pseudarthrosis site swab** |  |  |  | X |  |  |  |  |  |  |  |  |
| **ECG** |  | X |  |  |  |  |  |  |  |  |  |  |
| **X-rays** |  | X |  | X** |  | X | X | X | X | X | X | X |
| **Planning of surgery** |  | X |  |  |  |  |  |  |  |  |  |  |
| **Adipose tissue aspiration** |  |  | X |  |  |  |  |  |  |  |  |  |
| **Surgery for implantation of the cells** |  |  |  | x |  |  |  |  |  |  |  |  |
| **Safety evaluation** |  |  |  |  | X | X | X | X | X | X | X | X |
| **Pregnancy test** |  | X | X |  |  |  |  |  |  |  |  |  |

* The 24-month visit was carried out as safety control outside the setting of the clinical trial.

** Performed before the biograft product was implanted.

Abbreviations: ECG, electrocardiogram; ICF, informed consent form; m, month; V, visit; w, week

**Table S2.** Schedule of visits applicable to the patients treated with the allogenic biograft product.

|  | **Pre-inclusion** | **Pre-surgery** | **Surgery** | **Follow-up** | | | | | | |  |
| --- | --- | --- | --- | --- | --- | --- | --- | --- | --- | --- | --- |
|  |  | **V0**  **max. 60 d**  **before V1** | **V1**  **(+15-18 d)** | **V2** | **V3** | **V4** | **V5** | **V6** | **V7** | **V8** |  |
|  |  |  | **Surgery** | **1 w** | **1 m** | **3 m** | **4 m** | **6 m** | **9 m** | **12 m** | **24 m*** |
| **Patient information + ICF**  **Request for examinations** | X |  |  |  |  |  |  |  |  |  |  |
| **Incl. and excl. criteria** |  | X |  |  |  |  |  |  |  |  |  |
| **Medical history** |  | X |  |  |  |  |  |  |  |  |  |
| **Physical examination** |  | X |  | X | X | X | X | X | X | X | X |
| **Vital signs** |  | X |  | X | X | X | X | X | X | X | X |
| **Haematology & clinical biochemistry** |  | X |  | X |  |  |  |  |  | X | X |
| **Sample for serum bank** |  |  | X |  |  |  |  | X |  | X | X |
| **Pseudarthrosis site swab** |  |  | X |  |  |  |  |  |  |  |  |
| **ECG** |  | X |  |  |  |  |  |  |  |  |  |
| **X-rays** |  | X | X** |  | X | X | X | X | X | X | X |
| **Planning of surgery** |  | X |  |  |  |  |  |  |  |  |  |
| **Adipose tissue aspiration** |  |  |  |  |  |  |  |  |  |  |  |
| **Surgery for implantation of the cells** |  |  | X |  |  |  |  |  |  |  |  |
| **Safety evaluation** |  |  |  | X | X | X | X | X | X | X | X |
| **Pregnancy test** |  | X |  |  |  |  |  |  |  |  |  |

* The 24-month visit was carried out as safety control outside the setting of the clinical trial.

** Performed before the biograft product was implanted.

Abbreviations: ECG, electrocardiogram; ICF, informed consent form; m, month; V, visit; w, week

**Table S3.** Donor Inclusion and Exclusion Criteria.

|  | **INCLUSION CRITERIA** |
| --- | --- |
| 1 | Donor older than 18 years and younger than 60 |
| 2 | Patient information sheet |
| 3 | Informed consent form signed |
|  | **EXCLUSION CRITERIA** |
| 1 | HIV infected donor |
| 2 | Hepatitis B infected donor |
| 3 | Hepatitis C infected donor |
| 4 | Cytomegalovirus infected donor |
| 5 | Syphilis infected donor |
| 6 | HTLV-I/II infected donor |
| 7 | Biochemical test results outside normal range |
| 8 | Donor older than 61 years |
| 9 | Donor younger than 18 years |
| 10 | Donors with chronic or serious pathology |
| 11 | Donors with heart, blood pressure or blood flow pathology |
| 12 | Donors who have had a serious infectious disease (trypanosomiasis, malaria, leishmaniasis, mononucleosis) |

**Table S4.** Immunophenotypic and support adhesion characterization of batches used.

| **Patient** | **Batch** | **Immunophenotype** | | **Support adhesion (In process control)** |
| --- | --- | --- | --- | --- |
|  |  | **Specifications** | **Results** |  |
| 1-01-01 | I023 | CD13 positive ≥ 90%  CD44 positive ≥ 90%  CD73 positive ≥ 90%  CD90 positive ≥ 90%  CD105 positive ≥ 90%  CD14 negative ≥ 90%  CD19 negative ≥ 90%  CD34 negative ≥ 90%  CD45 negative ≥ 90%  HLA-DR negative ≥ 90% | 98.84%  98.62%  99.04%  98.46%  97.64%  100%  100%  99.98%  99.94%  99.99% | Complies |
|  | I025 |  | 98.85%  99.14%  97.47%  99.04%  95.87%  99.99%  100%  100%  100%  100% | Complies |
| 1-02-02 | J004 |  | 99.39%  99.09%  98.32%  98.84%  98.45%  100%  100%  100%  99.93%  99.95% | Complies |
| 1-04-03 | J005 |  | 99.09%  99.20%  98.39%  99.28%  98.18%  99.97%  100%  100%  100%  99.95% | Complies |
| 1-05-04 | K006 |  | 99.09%  99.20%  98.39%  99.28%  98.18%  99.97%  100%  100%  100%  99.95% | Complies |
| 1-06-05 | K010 |  | 99.3%  98.55%  97.9%  99.11%  97.69%  99.73%  99.99%  100%  99.98%  99.89% | Complies |
| 2-01-01 | J003 |  | 96.91%  98.70%  98.44%  99.50%  98.60%  99.98%  100%  100%  100%  99.98% | Complies |
| 1-07-06 | L007 |  | 98.27%  98.73%  98.51%  98.51%  98.52%  99.97%  99.92%  100%  100%  99.99% | Complies |
| 1-08-07 | L008 |  | 98.71%  98.51%  99.17%  99.35%  98.43%  99.92%  100%  99.99%  100%  99.91% | Complies |
| 1-11-08 | M001 |  | 97.33%  98.58%  99.21%  99.27%  98.96%  100%  100%  99.90%  100%  100% | Complies |
| 1-12-09 | M006 |  | 98.21%  99.10%  98.85%  97.64%  99.14%  99.96%  100%  100%  100%  100% | Complies |
| 1-14-10 | M013 |  | 96.88%  97.06%  96.88%  97.58%  96.93%  100%  99.99%  99.99%  100%  100% | Complies |
| 2-02-02 | L005 |  | 98.58%  98.62%  99.08%  98.33%  98.92%  100%  100%  100%  100%  99.99% | Complies |
|  | L006 |  | 98.75%  99.02%  99.13%  98.52%  99.03%  99.99%  100%  100%  100%  99.99% | Complies |

In the case of batches of allogeneic product, in addition to the previous tests, the cell differentiation study has been carried out in the Working Cell Bank (BCT) from which the finished product was manufactured:

- osteogenic differentiation
- chondrogenic differentiation
- adipogenic differentiation

**Table S5.** Cellular dose and biomaterial added per lesion volume.

|  | **Lesion volume (mL)** | | | | | | | | | | |
| --- | --- | --- | --- | --- | --- | --- | --- | --- | --- | --- | --- |
|  | **5** | **6** | **7** | **8** | **9** | **10** | **11** | **12** | **13** | **14** | **15** |
| **Cell dose (×10^6^)** | 15 | 18 | 21 | 24 | 27 | 30 | 33 | 36 | 39 | 42 | 45 |
| **Biomaterials** | 38 | 45 | 53 | 60 | 68 | 75 | 83 | 90 | 98 | 105 | 113 |

**Supplementary figures**

**Figure S1.** Syringe containing 10 CMT in a gelled medium (A). Each syringe provides a dose of 4±0.8 × 10^6^ AT-MSC. View of intraoperative application (B).

**A**


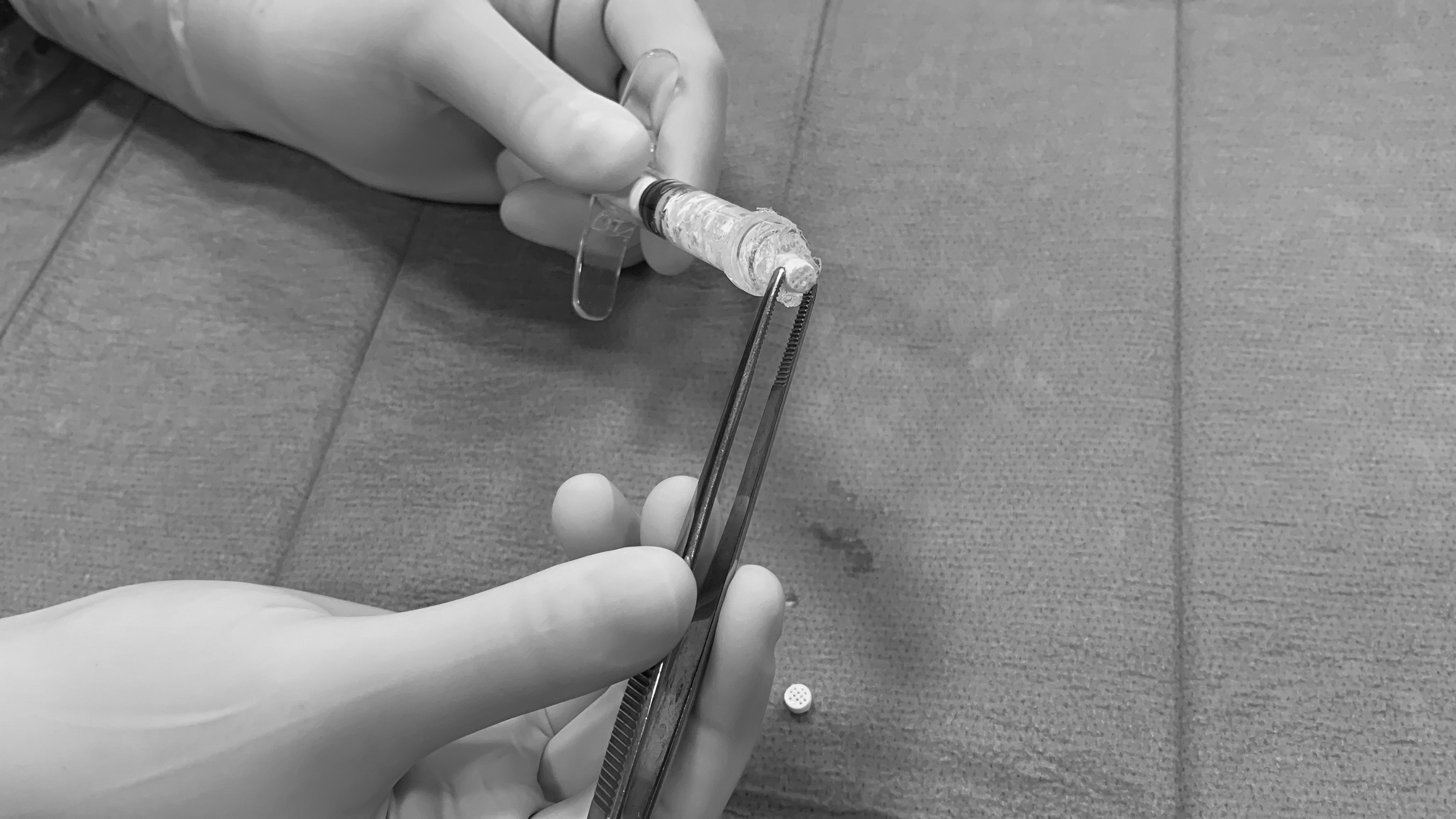


**B**


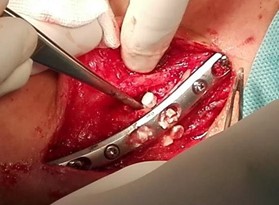


**Figure S2.** Scanning electron microscope analysis of the cellular response in the matrix over time.


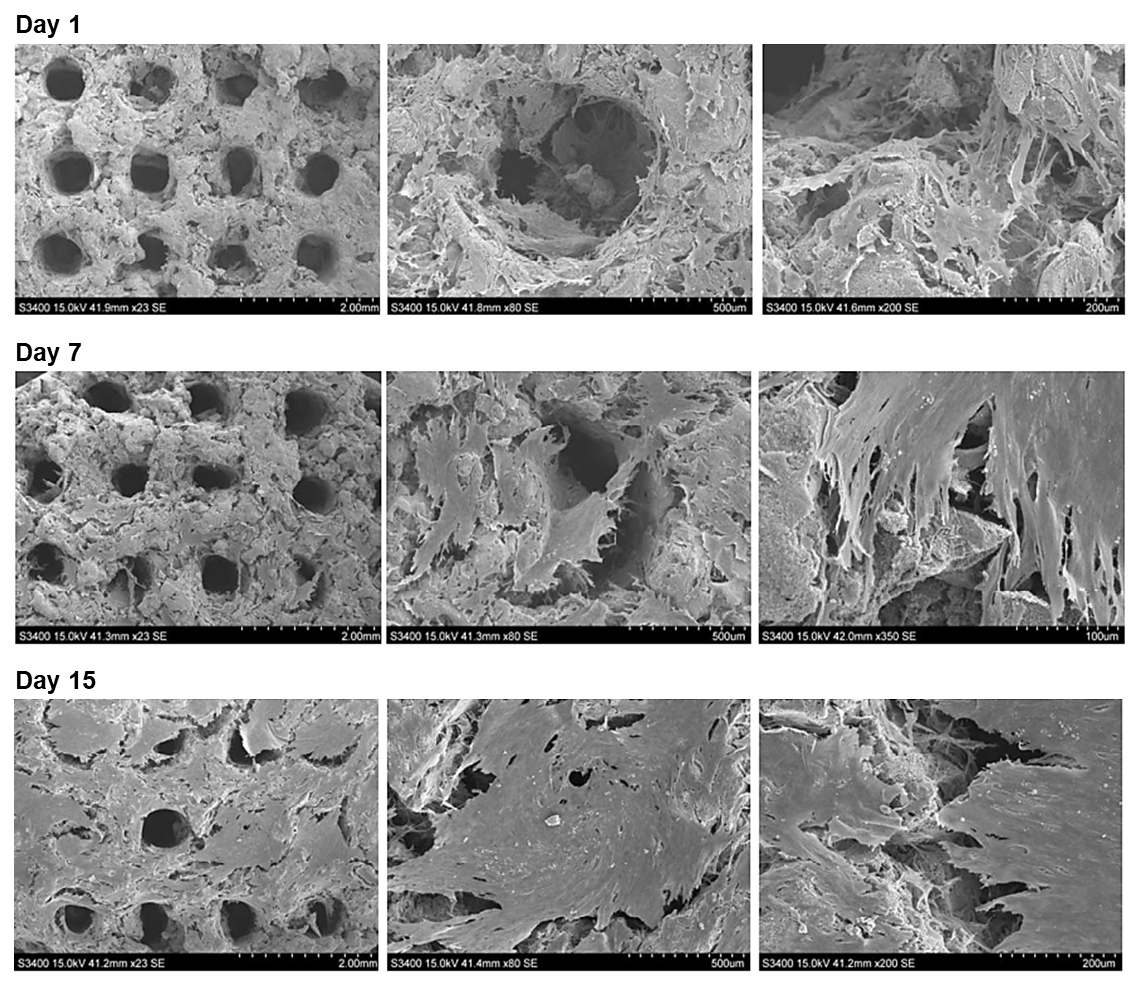

Supplement: Supplementary file 1 — Supplementary Material 1 [file 12967_2024_5280_MOESM1_ESM.docx]
